# Maternal circulating GPIHBP1 levels and neonatal outcomes in patients with gestational diabetes mellitus: a pilot study

**DOI:** 10.3389/fcdhc.2025.1682012

**Published:** 2025-10-10

**Authors:** Mayu Watanabe, Jun Eguchi, Naoko Kurooka, Eriko Eto, Hisashi Masuyama, Jun Wada

**Affiliations:** ^1^ Department of Diabetology and Endocrinology, National Hospital Organization (NHO) Okayama Medical Center, Okayama, Japan; ^2^ Department of Nephrology, Rheumatology, Endocrinology and Metabolism, Okayama University Faculty of Medicine, Dentistry and Pharmaceutical Sciences, Okayama, Japan; ^3^ Department of Obstetrics and Gynecology, Okayama University Faculty of Medicine, Dentistry and Pharmaceutical Sciences, Okayama, Japan

**Keywords:** glycosylphosphatidylinositol-anchored high-density lipoprotein-binding protein 1 (GPIHBP1), gestational diabetes mellitus (GDM), perinatal outcomes, placenta, triglyceride (TG)

## Abstract

**Introduction:**

The prevalence of gestational diabetes mellitus (GDM) is significantly increasing. Hyperglycaemia and dyslipidaemia have been demonstrated to contribute to endothelial dysfunction linked to foetal–placental circulation. Glycosylphosphatidylinositol-anchored high-density lipoprotein-binding protein 1 (GPIHBP1) is crucial for the lipolytic processing of TG-rich lipoproteins through the anchoring of lipoprotein lipase (LPL). In this study, circulating GPIHBP1 levels during pregnancy were evaluated, and their associations with hypertriglyceridaemia and the perinatal outcomes of GDM were evaluated.

**Methods:**

This study included 12 pregnant women with GDM and 21 pregnant women with normal glucose tolerance (NGT).

**Results:**

No significant differences in obstetrical outcomes were detected between the two groups. In participants with NGT, circulating GPIHBP1 levels were markedly lower in the 3rd trimester than in the 2nd trimester and at delivery. In women with GDM, circulating GPIHBP1 levels were unchanged during the 3rd trimester, and circulating GPIHBP1 levels throughout the 3^rd^ trimester were negatively correlated with neonatal birth weight percentile and umbilical venous pO_2_ (ρ=-0.636, p=0.026; ρ=-0.657, p=0.020).

**Discussion:**

Our findings suggest a possible association between circulating GPIHBP1 levels and perinatal outcomes in patients with GDM.

## Introduction

The current prevalence of gestational diabetes mellitus (GDM), which poses a significantly increased risk for perinatal complications, is notably high at 14% (1). Prepregnancy overweight or obesity and advanced maternal age have been identified as significant risk factors for GDM (2, 3). Foetal growth is influenced by maternal factors, including GDM, prepregnancy BMI, maternal age, and parity (4–6), and GDM is a well-established risk factor for large for gestational age (LGA) neonates, as affected women exhibit a 2.83-fold greater risk than those with normal glucose tolerance (7). Recent findings indicate that maternal glycaemic levels are not the sole risk factor for foetal overgrowth in cases of obesity and GDM (8). Certain studies have revealed an association between maternal blood triglyceride (TG) levels and neonatal weight, although no such correlation has been observed with maternal plasma glucose levels (9). In GDM, maternal blood TG levels are increased in early pregnancy compared with those in mothers without GDM and remain elevated throughout gestation, contributing to increased foetal subcutaneous fat mass and adiposity; maternal blood TG levels are also linked to foetal overgrowth (10, 11). Hyperglycaemia and dyslipidaemia have also been shown to contribute to the endothelial dysfunction associated with foetal–placental circulation (12–14).

Glycosylphosphatidylinositol-anchored high-density lipoprotein-binding protein 1 (GPIHBP1) is essential for the lipolytic processing of TG-rich lipoproteins (TRLs) because it anchors lipoprotein lipase (LPL) to the abluminal surface of blood capillaries, thereby stabilising its structure and facilitating its transport to the capillary lumen. GPIHBP1-anchored LPLs are crucial for the margination of TRLs within capillaries, which facilitates the process of lipolysis (15). Mutations in *GPIHBP1* have been associated with severe hypertriglyceridaemia, which results in an increased risk of acute pancreatitis, underscoring the importance of GPIHBP1 in intravascular TG processing (16). During pregnancy, women with *GPIHBP1* mutations exhibit high TG levels, particularly in the third trimester, which leads to severe pancreatitis and postnatal problems, including foetal distress (17, 18). Nonetheless, the relationships among circulating GPIHBP1 levels, dyslipidaemia, and maternal and foetal complications during GDM remain largely unexplored. This study assessed circulating GPIHBP1 levels during pregnancy and investigated their associations with hypertriglyceridaemia and perinatal outcomes in cases of GDM.

## Materials and methods

### Study participants

This prospective study included a cohort of 33 pregnant women recruited from the 26th of November, 2019, to the 31st of March, 2023. Participants were recruited from Okayama University Hospital, and GDM (n=12) was diagnosed using the 75 g oral glucose tolerance test (75 g OGTT) in accordance with the diagnostic criteria established by the International Association of Diabetes and Pregnancy Study Group (IADPSG) (19). Individuals with normal glucose tolerance (NGT, n=21) are defined as those who exhibit postprandial glucose levels below 100 mg/dL during screening tests or those who do not meet the diagnostic criteria for GDM after a 75 g OGTT. The exclusion criteria were as follows (1): multiple pregnancies (2); overt diabetes during pregnancy; and (3) preexisting type 1 or type 2 diabetes mellitus. Written informed consent was obtained from all participants. The study protocol received approval from the Ethics Committee of Okayama University (1910–015) and was executed in compliance with the Declaration of Helsinki.

### Data collection

Blood samples were collected after a 12-hour fast. Serum TG, HbA1c and glucose levels were quantified within one hour after blood collection by conventional methods using an automated clinical chemistry analyser (JCA-BM8040G; JEOL, Ltd., Tokyo, Japan). Serum samples were promptly frozen and stored at the Okayama University Hospital Biobank (Okadai Biobank) prior to the assessment of the other parameters using a GPIHBP1 (Immuno-Biological Laboratories [IBL]) enzyme-linked immunosorbent assay (ELISA) kits, as previously described (20).

Body mass index (BMI) was calculated using the following formula: body weight (kg)/height^2^ (m^2^). Systolic blood pressure was the median blood pressure recorded during the patient’s 5-day post-partum hospital stay. Medical history and current prescription information were extracted from each patient’s medical records.

### Histopathological examination of the placenta

The placenta was histologically examined by an experienced perinatal pathologist. Placental tissue samples were sliced into blocks of four μm, fixed in formalin, embedded in paraffin and stained with haematoxylin and eosin.

### Statistical analysis

Continuous variables are presented as the median (interquartile range: IQR), while categorical variables are expressed as absolute numbers or percentages. Differences between two groups in each separate experiment were analysed using Student’s *t* test, the nonparametric Mann–Whitney test, or the χ2 test. The Wilcoxon signed-rank test was employed to assess disparities between paired datasets. Spearman’s rank correlation was used to determine correlation coefficients. All the statistical analyses were conducted with SPSS Statistics version 25 (IBM Corp., Armonk, NY, USA). *P* values < 0.05 were considered to indicate statistical significance.

### *Post hoc* power analysis

To assess the reliability of the correlations in the GDM group (n = 12), *post hoc* power was calculated using G*Power 3.1.9.7 (Bivariate normal model, Exact test). In terms of the correlation between circulating GPIHBP1 levels and the neonatal birthweight percentile (ρ = -0.636) and between circulating GPIHBP1 levels and the umbilical venous pO_2_ level (ρ = -0.657), the observed power was approximately 0.65 and 0.70, respectively.

## Results

### Baseline characteristics and comparisons between study groups

In all, 12 women with GDM and 21 participants with NGT, all of whom were Japanese, were included in this study. Blood samples were obtained during the 2^nd^ trimester at 25 to 26 weeks of gestation, during the 3^rd^ trimester at 35 to 36 weeks and within 3 days after delivery. **Table 1** describes the baseline characteristics and comparisons between the study groups. Statistical analysis indicated that compared with participants with NGT, women with GDM had significantly higher pregestational BMI and HbA1c levels (in the 3^rd^ trimester and at delivery). Women with GDM had significantly lower gestational weight gain (GWG), total cholesterol (3^rd^ trimester), and HDL (3^rd^ trimester) levels than did participants with NGT. Although women with GDM received dietary counselling only once at the initial visit, they exhibited lower gestational weight gain than did women with NGT, which likely reflects dietary glycaemic management and efforts to control weight. Nonetheless, no statistically significant difference was observed between the two groups in terms of age, proportion of primiparous women, total cholesterol (at delivery), LDL cholesterol (3^rd^ trimester and at delivery), HDL cholesterol (at delivery), LDL-cholesterol/HDL-cholesterol (3^rd^ trimester and at delivery), or TG levels (3^rd^ trimester and at delivery).

**Table 1:** Characteristics of the GDM and NGT groups.

Variables		GDM	NGT	p value
		(n=12)	(n=21)	
Age (years)		35 (33–40)	36 (32.5-39.5)	0.956
Primipara, n (%)		9 (75)	12 (57.1)	0.457
Pregestational BMI (kg/m^2^)		26.4 (23.6-30.9)	20.6 (19.6-22.6)	**0.001**
GWG (kg)		3.9 (-0.0-6.4)	8.1 (5.4-9.2)	**0.004**
HbA1c (%)	3^rd^ trimester	5.8 (5.5-6.0)	5.5 (5.4-5.8)	**0.033**
	delivery	5.7 (5.0-6.1)	5.4 (5.0-5.5)	**0.049**
Total cholesterol (mg/dL)	3^rd^ trimester	256 (204-280)	296 (264-342)	**0.018**
	delivery	222 (172-239)	236 (211-274)	0.131
LDL cholesterol (mg/dL)	3^rd^ trimester	147 (85-164)	170 (136-207)	0.082
	delivery	117 (85-133)	134 (109-156)	0.213
HDL cholesterol (mg/dL)	3^rd^ trimester	61 (52-83)	78 (72-88)	**0.030**
	delivery	55 (43-67)	64 (53-72)	0.069
LDL-cholesterol/HDL-cholesterol	3^rd^ trimester	2.0 (1.5-2.5)	2.2 (1.8-2.4)	0.618
	delivery	2.1 (1.6-2.4)	2.1 (1.8-2.4)	0.897
TG (mg/dL)	3^rd^ trimester	279 (192-338)	338 (215-389)	0.345
	delivery	246 (167-355)	204 (145-252)	0.308

Data are presented as medians (25–75th percentile) for continuous variables and as percentages for categorical variables.

GDM, gestational diabetes mellitus; NGT, normal glucose tolerance; BMI, body mass index; GWG, gestational weight gain; HbA1c, glycated haemoglobin; LDL, low‐density lipoprotein; HDL, high‐density lipoprotein.

Values in bold indicate statistically significant differences (P < 0.05).

### Comparisons of obstetrical outcomes and neonatal characteristics between study groups

With respect to obstetrical outcomes, no significant differences were observed between the two groups in terms of gestational age at delivery and in the incidences of preterm delivery, emergency caesarean section, or hypertensive disorders of pregnancy (HDP) (**Table 2**). In terms of neonatal characteristics, the two groups did not significantly differ in terms of neonatal birth weight, LGA, small for gestational age (SGA), Apgar score (AS), neonatal plasma glucose, umbilical blood gas analysis, or incidence of foetal distress (**Table 2**).

**Table 2:** Obstetrical outcomes and neonatal characteristics of the GDM and NGT groups.

Variables	GDM	NGT	p value
	(n=12)	(n=21)	
Obstetrical outcomes			
Gestational age at delivery (weeks)	39 (38-40)	39 (38-39)	1.000
Preterm delivery, n (%)	0 (0)	0 (0)	–
Emergency caesarean section, n (%)	0 (0)	2 (9.5)	0.523
Systolic BP	118 (111-122)	111 (106-120)	0.242
HDP, n (%)	2 (16.7)	2 (9.5)	0.610
Neonatal characteristics			
Neonatal birth weight (g)	3056 (2904-3337)	3122 (2703-3236)	0.811
Neonatal birth weight percentile	43.0 (34.2-76.3)	57.8 (28.5-77.4)	0.985
LGA	2 (16.7)	3 (14.3)	1.000
SGA	0 (0)	1 (4.8)	1.000
AS 1 min	8 (8-8)	8 (8-8)	0.699
AS 5 min	9 (9-9)	9 (9-9)	1.000
Neonatal plasma glucose (mg/dL)	61 (47-74)	63 (52-70)	0.860
UmApH	7.31 (7.28-7.33)	7.30 (7.25-7.34)	0.927
UmApO_2_	17.4 (14.2-21.3)	19.1 (16.3-23.7)	0.408
UmVpH	7.35 (7.32-7.38)	7.34 (7.31-7.35)	0.477
UmVpO_2_	27.6 (22.6-32.2)	24.9 (21.9-32.8)	0.632
Foetal distress	0 (0)	0 (0)	–

BP, blood pressure; HDP, hypertensive disorders of pregnancy; LGA, large for gestational age; SGA, small for gestational age; AS, Apgar score; UmApH, umbilical artery pH; UmApO_2_, umbilical artery pO_2_; UmVpH, umbilical venous pH; UmVpO_2_, umbilical venous pO_2_.

### Changes in circulating GPIHBP1 levels during pregnancy

**Figure 1:**
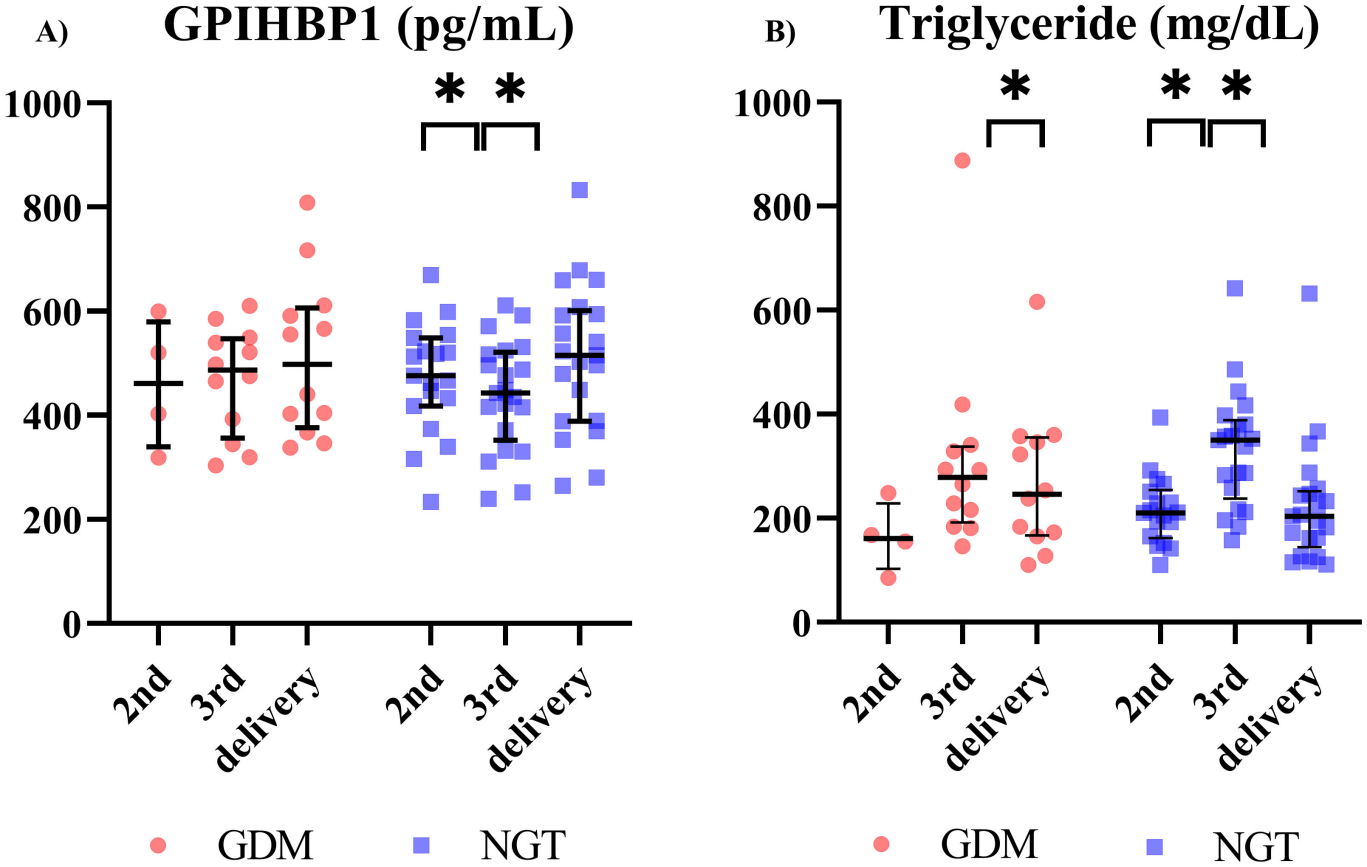
Longitudinal changes in circulating GPIHBP1 and triglyceride (TG) levels during pregnancy in women with gestational diabetes mellitus (GDM) and participants with normal glucose tolerance (NGT) (GDM; n=12; NGT; n=21). **(A)** Circulating GPIHBP1 levels. **(B)** Maternal TG levels. Red circles indicate women with GDM, and blue squares indicate women with NGT. The data are presented as individual values. Wilcoxon signed-rank test; *p < 0.05.

Next, we evaluated circulating GPIHBP1 levels during pregnancy in participants with NGT. Circulating GPIHBP1 levels were markedly lower in the 3^rd^ trimester than in the 2^nd^ trimester and at delivery (**Figure 1A**). Conversely, serum TG levels had markedly increased in the 3^rd^ trimester compared with the 2^nd^ trimester and at delivery (**Figure 1B**). In women with GDM, circulating GPIHBP1 levels and serum TG levels were unchanged during the 3^rd^ trimester (**Figure 1**). circulating GPIHBP1 levels during the third trimester were not significantly correlated with serum TG levels. Circulating GPIHBP1 levels throughout the 3^rd^ trimester were not significantly correlated with serum TG levels in both the NGT and GDM groups.

### Correlation between circulating GPIHBP1 levels and perinatal outcomes of patients with GDM

**Figure 2:**
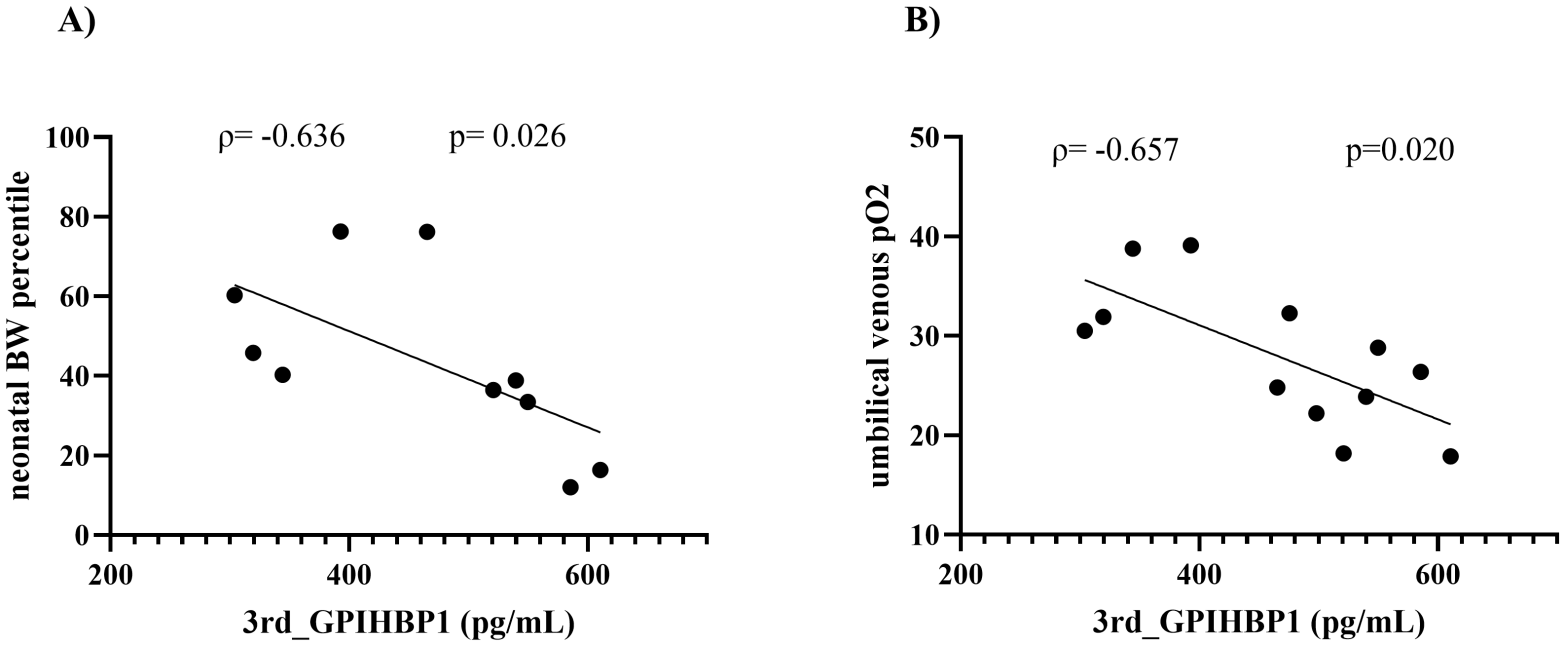
Correlations between circulating GPIHBP1 levels and neonatal outcomes in women with GDM. Spearman’s rank correlation coefficients (ρ) and corresponding p values are indicated for each relationship. **(A)** Correlations between circulating GPIHBP1 levels in the 3^rd^ trimester and neonatal birth weight (BW) percentiles. **(B)** Correlations between circulating GPIHBP1 levels in the 3^rd^ trimester and umbilical venous pO_2_ levels.

Given the variability in circulating GPIHBP1 levels during the 3^rd^ trimester, we investigated the correlation between circulating GPIHBP1 levels and perinatal complications during the 3^rd^ trimester. In the 3^rd^ trimester, circulating GPIHBP1 levels were negatively correlated with neonatal birth weight (BW) percentile(p=-0.636, p=0.026) (**Figure 2A**). Furthermore, circulating GPIHBP1 levels throughout the 3^rd^ trimester were negatively correlated with umbilical venous pO_2_ levels (ρ=-0.657; p=0.020) (**Figure 2B**). After the Bonferroni correction was applied for multiple testing (α = 0.025), only the correlation with umbilical venous pO_2_ remained statistically significant. Notably, TG levels throughout the 3^rd^ trimester were positively correlated with maternal age and prepregnancy BMI (maternal age: ρ=0.647, p=0.023; prepregnancy BMI: ρ=0.629, p=0.028); however, no association was observed with neonatal outcome.

In this study, among six women with placental pathology, circulating GPIHBP1 levels throughout the 3^rd^ trimester were elevated in those with placental infarction (n=2) and in those with chorangiosis (n=1) compared with those without these conditions (n=3). However, these observations are exploratory given the very limited sample size (**Supplementary Figures 1, 2**).

Additionally, maternal age, maternal BMI, GWG, HbA1c (3^rd^ trimester), LDL-C (3^rd^ trimester) and HDL-C (3^rd^ trimester) were not significantly associated with GPIHBP1 levels, neonatal birth weight percentiles or umbilical venous pO_2_ in this cohort. Furthermore, no significant differences in neonatal BW percentile or umbilical venous pO_2_ levels were observed between the groups in patients who met one positive criterion and in those who met two or 3 positive criteria on the 75 g OGTT (neonatal BW percentile: p=1.000; umbilical venous pO_2_ level: p=0.527).

## Discussion

In this study, we demonstrated that circulating GPIHBP1 levels were markedly lower in the 3^rd^ trimester than in the 2^nd^ trimester and at delivery. Notably, circulating GPIHBP1 levels in women with GDM were negatively correlated with neonatal BW percentiles (ρ = -0.636, p = 0.026) and umbilical venous pO_2_ (ρ = -0.657, p = 0.020). In contrast, maternal TG levels throughout the 3^rd^ trimester were not associated with neonatal outcomes. This work represents a novel contribution, as it is the first to suggest a potential link between circulating GPIHBP1 levels and perinatal outcomes.

Previous studies have indicated that GDM is associated with an increased risk of perinatal complications such as macrosomia, preterm birth, polyhydramnios, and preeclampsia (3, 7) and have reported that pregnant women with GDM or obesity typically exhibit higher TG levels than do women of normal weight (10). However, in our study, TG levels in women with GDM did not differ significantly from those in women with NGT, and perinatal outcomes also did not differ markedly between the groups. Previous studies have indicated that elevated serum TG levels during pregnancy are associated with an increased risk of higher birth weight (21, 22), which results in excessive fat accumulation in the foetus (23). Early maternal obesity has been reported to be a risk factor for neonatal adiposity (10, 24). Even in well-managed GDM pregnancies, maternal TG levels remain strong predictors of foetal lipid profiles and foetal growth (25). Despite these findings, in the present study, maternal TG levels throughout the 3^rd^ trimester were not associated with the neonatal BW percentile. In healthy pregnant women, total cholesterol, TG, and HDL-cholesterol levels typically increase during pregnancy, while the atherogenic index (LDL-cholesterol/HDL-cholesterol) remains unchanged. In contrast, women with GDM exhibit elevated TG levels, altered cholesterol and lipoprotein levels, and reduced HDL levels (9). In the present study, women with GDM exhibited lower HDL-C levels, whereas TG levels and the atherogenic index did not differ significantly from those in women with NGT, which suggests that lipid metabolic alterations in women with GDM in the present cohort may have been less pronounced than those reported in previous studies. The women with GDM in this cohort had a median prepregnancy BMI of 26.4 kg/m²; these women were categorised as overweight, and their median GWG was only 3.9 kg, which is below the recommended range of 6.8–11.3 kg for this population (26). Insufficient GWG has been linked to an increased risk of SGA (27), which may partly explain the lack of association between maternal TG levels and neonatal birthweight percentiles observed in this study.

Currently, no findings have been published on GPIHBP1 or its role in placental function, and the regulatory mechanisms underlying circulating GPIHBP1 levels during pregnancy remain to be elucidated. In this study, circulating GPIHBP1 levels in women with GDM tended to be negatively correlated with neonatal birth weight percentiles, regardless of maternal TG levels. Although GDM is typically linked to excessive foetal growth and consequently higher rates of LGA and macrosomia, associations with SGA have also been reported, which suggests that GDM affects foetal growth through a variety of mechanisms (28). GPIHBP1 is essential for TG hydrolysis because it binds LPL and facilitates its transit from the extravascular space to the lumen (15). GPIHBP1 is expressed predominantly in adipose tissue, but its expression has also been documented in the placenta (29). LPL mRNA is expressed on both the maternal and foetal sides of the human placenta, and the LPL protein is also detectable in foetal endothelial cells. In other tissues, such as adipose tissue and skeletal muscle, parenchymal adipocytes and myocytes produce LPL, which is subsequently transported to the luminal surface of the vascular endothelium. LPL hydrolyses TG-rich lipoproteins to generate free fatty acids for uptake by local tissues. LPL in placental endothelial cells may facilitate the uptake of lipids from both the maternal circulation and the foetal circulation, thereby contributing to the foetal nutrient supply (30). In pregnant rats, LPL activity decreases in adipose tissue and the liver during late pregnancy but increases in the placenta. In the placenta, TG of maternal origin is hydrolysed into free fatty acids, which are supplied to the foetus (31). These observations highlight the substantial alterations in lipid metabolism that occur during the 3^rd^ trimester. In this context of marked metabolic changes, LPL anchored by GPIHBP1 may be modulated, which may affect circulating GPIHBP1 levels. Notably, even within a range of TG changes that did not differ significantly from those in women with NGT, circulating GPIHBP1 levels tended to be negatively correlated with neonatal birth weight percentiles, which suggests that circulating GPIHBP1 may reflect aspects of foetal growth regardless of maternal TG levels.

In the present study, circulating GPIHBP1 levels tended to be negatively correlated with umbilical venous pO_2_ in women with GDM. Umbilical vein blood gas analysis primarily indicates placental metabolism (32). Placental dysfunction is associated with complications, including low birth weight in infants (33, 34). Our prior work indicated that circulating GPIHBP1 levels are associated with the incidence of microvascular complications in women with type 2 diabetes, irrespective of TG levels (35). Comparable results were reported in a study that investigated the association between vascular disorders and circulating GPIHBP1 levels (20). Circulating GPIHBP1 levels may be associated with vascular damage irrespective of serum TG levels. Previous studies have also suggested an association between GDM and SGA, which may reflect vascular or endothelial dysfunction and placental insufficiency, suggesting that multiple mechanisms may underlie the influence of GDM on foetal growth (28). In pregnancies complicated by GDM and/or maternal overweight or obesity, fetoplacental endothelial dysfunction appears to arise from dysfunction in the regulation of several critical pathways, including epigenetic modifications, inflammatory signalling, nitric oxide-mediated vascular signalling, mitochondrial function, and alterations in the L-arginine/nitric oxide and insulin/adenosine signalling axes (36, 37). GPIHBP1 is localised to endothelial cells, and fetoplacental endothelial dysfunction could be related to its expression and possibly to its levels in the circulation. Although the sample size was limited, circulating GPIHBP1 levels tended to be elevated in patients with placental infarction and in those with chorangiosis. Previous studies have suggested that placental infarction, a marker of maternal vascular insufficiency in placental pathology, is associated with foetal growth failure (38), whereas chorangiosis, a vascular alteration affecting the terminal villi of the placenta, arises from moderate hypoxia and is linked to intrauterine growth restriction (39). Taken together, the observed negative correlation between circulating GPIHBP1 levels and umbilical venous pO_2_ in GDM pregnancies suggests that circulating GPIHBP1 may serve as an indicator of placental vascular function, particularly given the multifaceted involvement of GDM and prepregnancy overweight or obesity in vascular dysfunction. Several biomarkers have already been proposed to reflect placental dysfunction (40, 41); our findings indicate that circulating GPIHBP1 may also partially reflect placental function, which highlights its potential as a novel biomarker.

Despite our novel findings, this study has several limitations. First, the small sample size inherent to this pilot study constitutes a major limitation. The limited cohort reduces statistical power and considerably restricts the generalisability of the findings; therefore, the results should be interpreted with caution. Confirmation in larger, independent cohorts is essential. Second, heterogeneity was observed in the interventions: the GDM group received a single session of dietary counselling, while the NGT group received no intervention. Given that the intervention was limited to one session, its direct impact was likely minimal. The observed differences may instead reflect heightened individual attention to gestational weight control in the GDM group, which could not be quantified. This represents an additional important limitation when interpreting the results. Third, multiple correlations were conducted without formal adjustment for multiple testing. After the Bonferroni correction was applied, only the correlation between circulating GPIHBP1 levels and umbilical venous pO_2_ remained statistically significant. Given the limited number of cases, these findings should be interpreted with caution, as the correction may have been overly conservative. Although maternal BMI, maternal age, and parity were not significantly associated with GPIHBP1 levels, birth weight percentile, umbilical venous pO_2_, neonatal blood glucose, or umbilical arterial pO_2_ in our exploratory analyses, the small sample size limits the statistical power to exclude residual confounding. Finally, the impact of GPIHBP1 on placental function remains underexplored, and thus additional comprehensive pathological and molecular biological investigations are needed. Taken together, these limitations should be carefully considered when interpreting the results.

In summary, our study suggests that in women with GDM, higher circulating GPIHBP1 levels may be associated with lower birth weight percentiles and lower umbilical venous pO_2_ levels. These observations may indicate a potential association between circulating GPIHBP1 levels and placental function; however, this notion remains preliminary and requires confirmation in future studies.

## Data Availability

The raw data supporting the conclusions of this article will be made available by the authors, without undue reservation.
