# Correction: Maternal circulating GPIHBP1 levels and neonatal outcomes in patients with gestational diabetes mellitus: a pilot study

**DOI:** 10.3389/fcdhc.2025.1724699

**Published:** 2025-10-27

**Authors:** Mayu Watanabe, Jun Eguchi, Naoko Kurooka, Eriko Eto, Hisashi Masuyama, Jun Wada

**Affiliations:** ^1^ Department of Diabetology and Endocrinology, National Hospital Organization (NHO) Okayama Medical Center, Okayama, Japan; ^2^ Department of Nephrology, Rheumatology, Endocrinology and Metabolism, Okayama University Faculty of Medicine, Dentistry and Pharmaceutical Sciences, Okayama, Japan; ^3^ Department of Obstetrics and Gynecology, Okayama University Faculty of Medicine, Dentistry and Pharmaceutical Sciences, Okayama, Japan

**Keywords:** glycosylphosphatidylinositol-anchored high-density lipoprotein-binding protein 1 (GPIHBP1), gestational diabetes mellitus (GDM), perinatal outcomes, placenta, triglyceride (TG)

The order of authors in the author list of the published paper was erroneously given as:

“Mayu Watanabe^1,2^*, Jun Wada^2^, Naoko Kurooka^2^, Eriko Eto^3^, Hisashi Masuyama^3^, Jun Eguchi^2^”

The correct author list reads:

“Mayu Watanabe^1,2^*, Jun Eguchi^2^, Naoko Kurooka^2^, Eriko Eto^3^, Hisashi Masuyama^3^, Jun Wada^2^”

The **Author Contributions** statement has been corrected to read:

“MW: Data curation, Conceptualization, Investigation, Methodology, Writing – review & editing, Writing – original draft, Formal Analysis. JE: Supervision, Writing – review & editing, Writing – original draft. NK: Supervision, Data curation, Writing – review & editing. EE: Supervision, Data curation, Writing – review & editing. HM: Supervision, Data curation, Writing – review & editing. JW: Writing – original draft, Supervision, Writing – review & editing.”

The original version of this article has been updated.

