# Supplementary figures and images for: Maternal circulating GPIHBP1 levels and neonatal outcomes in patients with gestational diabetes mellitus: a pilot study
Source: Front Clin Diabetes Healthc. 2025 Oct 10;6:1682012. doi: 10.3389/fcdhc.2025.1682012 (PMC12549623; doi:10.3389/fcdhc.2025.1682012)

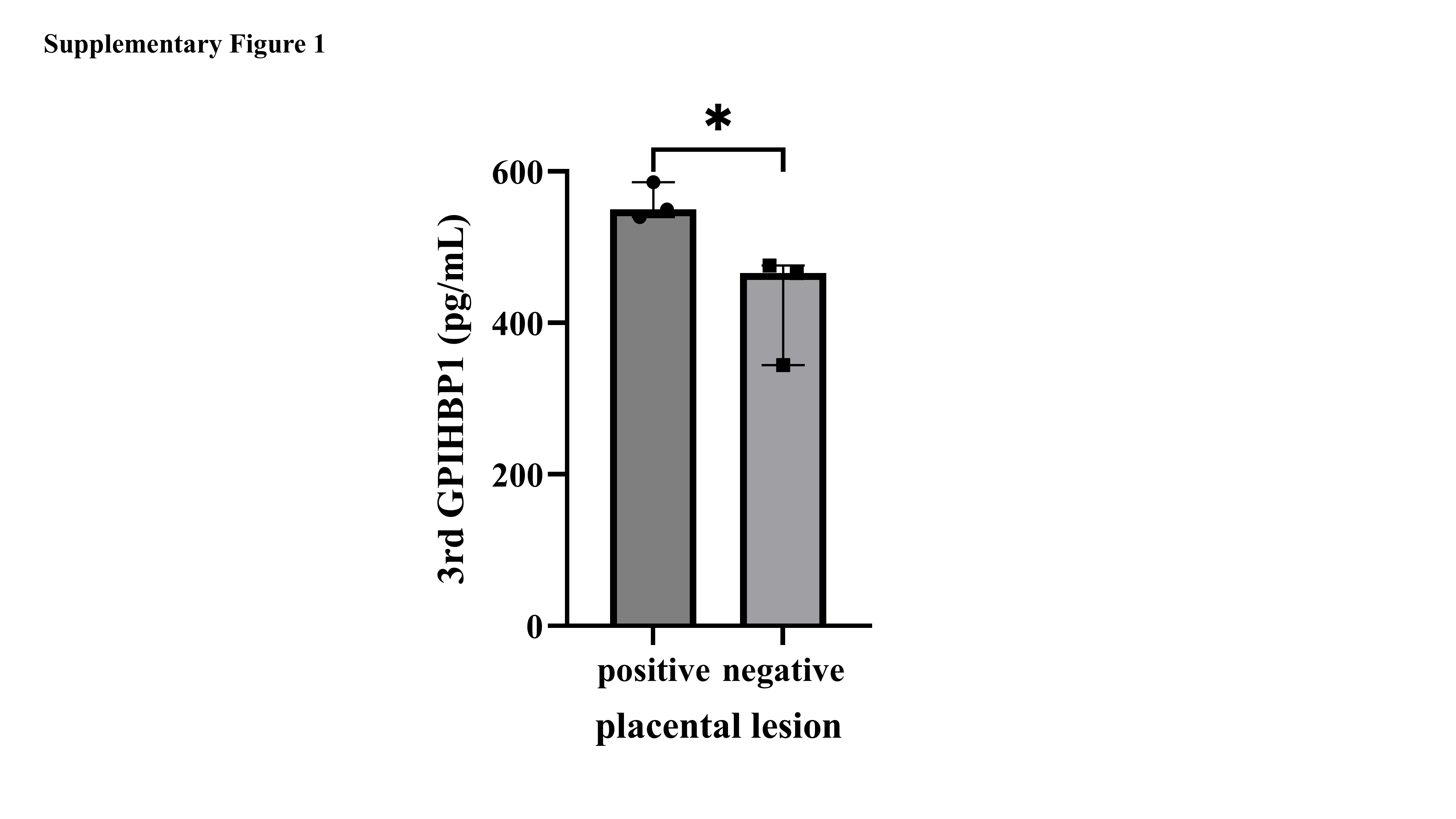

Supplement: Supplementary file 1 [file Image1.tif]

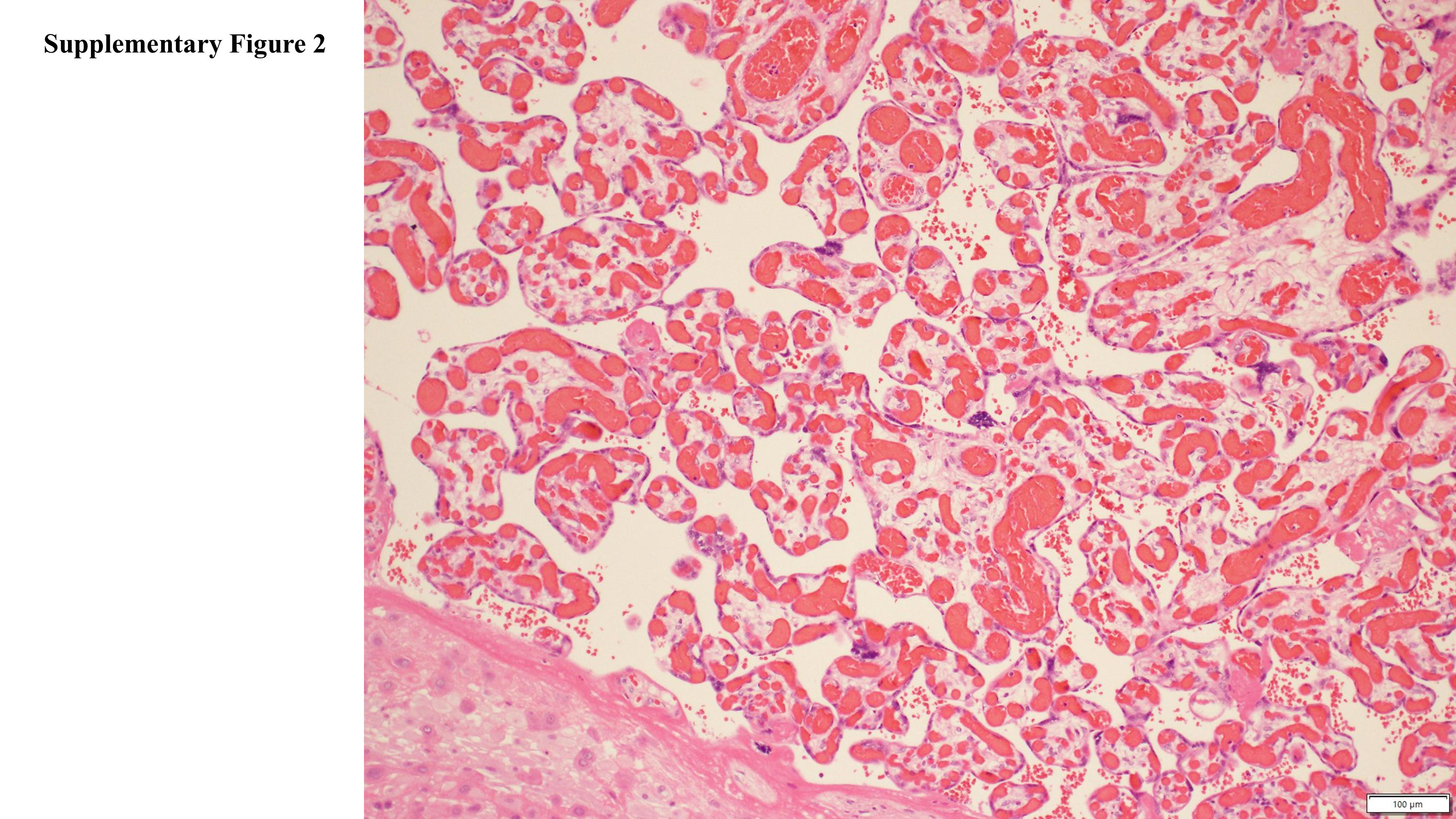

Supplement: Supplementary file 2 [file Image2.tif]
